# Intensive care unit versus high-dependency care unit admission on mortality in patients with septic shock: let’s think to the survival chain concept for septic shock

**DOI:** 10.1186/s40560-022-00643-2

**Published:** 2022-12-05

**Authors:** Romain Jouffroy, Papa Gueye

**Affiliations:** 1 Intensive Care Unit, Ambroise Paré University Hospital, Assistance Publique-Hôpitaux de Paris, and Paris Saclay University, Paris, France; 2 SAMU 972 University Hospital of Martinique, Fort-de-France, Martinique France

To the editor,

We read with great interest the recent article published in the Journal by Endo et al. [1], reporting a positive association between intensive care unit (ICU) compared to high-dependency care units (HDUs) admission and lower 30-day mortality in patients with septic shock.

The authors must be congratulated for these very interesting findings in line with international sepsis guidelines to develop a more appropriate treatment system [2–4]. Beyond the prompt recognition and the severity assessment of sepsis prior to treatments implementation [2–4], admission to adequate facility to reduce sepsis mortality rate, especially for the sicker ones and the most frail patients, i.e., septic shock, appears to be essential as pointed by Endo et al. [1]. For septic shock patients, the in-hospital “bundle of care” completion, associated with outcome improvement [5], from sepsis detection to treatment delivery requires the presence of a sufficient number of qualified caregivers. In Endo et al. study [1], in HDUs, the patient–nurse ratio is two times lower than in ICU and no full-time physician is needed; thus we can suppose that the delays for severity assessment and treatments initiation are probably longer than for patients admitted to ICU, despite Endo et al. [1] study design does not allow this conclusion. Moreover, we cannot exclude the contribution of an influence of patient recruitment volume on the outcome as previously reported for sepsis, subarachnoid hemorrhage and ECMO [6–8]. This is in line with the “bundle of care” concept stressing that not a single treatment alone can improve outcome, but the combination of different treatments; in other words, therapeutic strategies including organizational considerations. Similarly to cardiac arrest and post-cardiac arrest management [9], we believe that there is a need for a specific chain of survival for sepsis, especially for septic shock, started since the prehospital setting to decrease sepsis-related mortality. Beyond early identification and sepsis severity assessment, antibiotic therapy and early hemodynamic optimization are the main elements associated with an increased survival of septic shock patients cared for in the prehospital setting [10–12].

Further prospective studies are needed to clarify the real treatment effect of immediate ICU admission for patients suffering from septic shock.

## Data Availability

Not applicable.
